# Heuristic Approaches for Coordinating Collaborative Heterogeneous Robotic Systems in Harvesting Automation with Size Constraints

**DOI:** 10.3390/s25206443

**Published:** 2025-10-18

**Authors:** Hyeseon Lee, Jungyun Bae, Abhishek Patil, Myoungkuk Park, Vinh Nguyen

**Affiliations:** 1 Department of Mechanical and Aerospace Engineering, Michigan Technological University, Houghton, MI 49931, USA; hyeseonl@mtu.edu (H.L.); apatil5@mtu.edu (A.P.); mkpark@mtu.edu (M.P.); vinhn@mtu.edu (V.N.); 2 Department of Applied Computing, Michigan Technological University, Houghton, MI 49931, USA

**Keywords:** multi-robot systems, agricultural automation, task allocation, path planning

## Abstract

Multi-agent coordination with task allocation, routing, and scheduling presents critical challenges when deploying heterogeneous robotic systems in constrained agricultural environments. These systems involve real-time sensing during their operations with various sensors, and having quick updates on coordination based on sensed data is critical. This paper addresses the specific requirements of harvesting automation through three heuristic approaches: (1) primal–dual workload balancing inspired by combinatorial optimization techniques, (2) greedy task assignment with iterative local optimization, and (3) LLM-based constraint processing through prompt engineering. Our agricultural application scenario incorporates robot size constraints for navigating narrow crop rows while optimizing task completion time. The greedy heuristic employs rapid initial task allocation based on proximity and capability matching, followed by iterative route refinement. The primal–dual approach adapts combinatorial optimization principles from recent multi-depot routing solutions, dynamically redistributing workloads between robots through dual variable adjustments to minimize maximum completion time. The LLM-based method utilizes structured prompt engineering to encode spatial constraints and robot capabilities, generating feasible solutions through successive refinement cycles. We implemented and compared these approaches through extensive simulations. Preliminary results demonstrate that all three approaches produce feasible solutions with reasonable quality. The results demonstrate the potential of the methods for real-world applications that can be quickly adopted into variations of the problem to offer valuable insights into solving complex coordination problems with heterogeneous multi-robot systems.

## 1. Introduction

The automation of agricultural harvesting processes through collaborative heterogeneous robotic systems represents a significant advancement in addressing global challenges such as food security, labor shortages, and sustainable farming practices, particularly for small farms. Small-scale farms, which often prioritize artisanal products or small-scale distillations, face acute labor shortages and cannot afford the specialized machinery used by larger operations. The potential for the automation of small farm harvesting through adaptive collaboration with heterogeneous robotic fleets is considerable. However, the effective deployment of such systems requires solving complex path planning problems that extend beyond traditional optimization challenges.

Agricultural automation presents unique challenges due to several factors. A farm may cultivate multiple crop varieties, each requiring different harvesting techniques or handling, thus necessitating a fleet of heterogeneous robots with specialized capabilities. As a case study, consider a lavender farm where different species require robots of varying sizes and toolsets for optimal harvesting. Additionally, the dynamic nature of the agricultural environment, with seasonal and daily changes in field conditions, necessitates optimizing for efficiency while satisfying diverse robot-specific constraints such as size, payload capacity, and fuel consumption. Each farm has different restrictions and needs, which may require different strategies for collaborative operations. Our research aims to develop algorithms for vehicle routing problems under diverse settings with generalized cases applicable to various domains beyond agriculture, such as warehouse logistics, surveillance, and search and rescue operations [1,2,3,4,5,6,7,8].

Recent research has proposed various state-of-the-art techniques for agricultural automation involving multiple heterogeneous robots. For instance, Conesa-Muñoz et al. introduced a simulated annealing-based method for path planning of heterogeneous agricultural vehicles, optimizing criteria such as distance, cost, and time while considering fuel capacity [9] under the simplified assumptions of uniform field geometry. Furchì et al. developed a route planning strategy for heterogeneous mobile robots in precision agriculture, utilizing a multi-Steiner Traveling Salesman Problem to optimize task assignments and paths while accounting for fuel constraints [10]. Mukhamediev et al. proposed a genetic-algorithm-based method for coverage path planning of heterogeneous aerial vehicles with fuel constraints, focusing on environments with convex fields without obstacles [11]. Urvina et al. presented an integrated planning strategy for Skid–Steer Mobile Robots in complex agricultural environments, combining a global planning algorithm with a local path planner based on the Informed Rapidly exploring Random Tree Star [12]. This method prioritizes harvesting positions based on criteria such as minimum path length and vehicle load capacity while considering motion constraints. Pak et al. evaluated path planning algorithms for smart farms, concluding that the A* algorithm is suitable for minimizing crop damage and demonstrates high localization accuracy, although their study was limited to single-robot scenarios [13]. Utamima and Reiners addressed Agricultural Route Planning in multifield environments with irregular field shapes, aiming to optimize paths for (semi-)autonomous machines using a Fast Hybrid Algorithm [14].

Although these approaches offer valuable insights, they are often tailored to specific applications, which can limit their extensibility. This specialization highlights the need for more versatile and adaptable solution frameworks. Our work explicitly integrates robot-specific size constraints at narrow passages into a generalized framework and introduces a novel Large Language Model (LLM)-based heuristic for task allocation and path planning without task-specific training. This research develops and evaluates three distinct heuristic approaches: (1) a primal–dual heuristic derived from the problem’s mathematical formulation; (2) a fast greedy heuristic based on intuitive, iterative improvement; and (3) the LLM-based heuristic that leverages advanced prompt engineering. Our primary objective is to minimize the makespan (total completion time) while adhering to robot-specific size constraints at narrow passages. By developing and comparing these diverse methods, this paper provides a comprehensive analysis of different strategies for tackling complex coordination challenges. This approach not only addresses the immediate needs of agricultural automation but also offers insights with implications for a wide range of applications beyond agriculture.

## 2. Problem Description and Formulation

**Figure 1:**
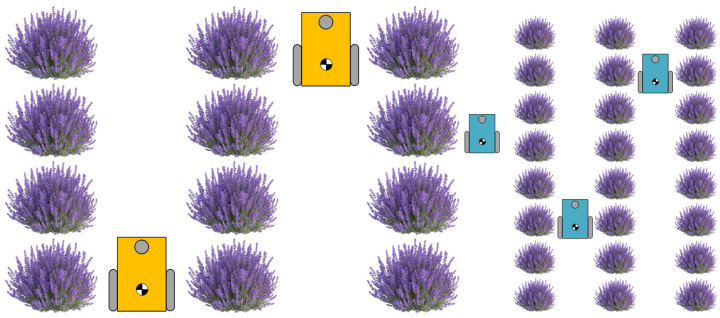
An example of the proposed problem with two different-sized robots navigating size-constrained paths in a lavender farm.

We are given a set of heterogeneous robots, each with a unique depot location, speed, physical size, and a set of tasks to be completed. The goal is to design a route for each robot that enables it to complete all tasks. The solution must be optimized for the following objective and adhere to a specific set of constraints. (1) Task coverage: Ensure that at least one robot handles every task. (2) Motion constraints: Each route must satisfy the motion constraints specific to the corresponding robot, accounting for its unique capabilities and limitations. (3) Size constraints: Each route must satisfy the size constraints for each robot, which means the robot is only allowed to travel in passages wider than its width. (4) Minimized job completion time: We aim to find the most efficient route for each robot to complete all tasks while minimizing the maximum operating time among all the robots. Figure 1 illustrates a sample scenario in a lavender farm, where two robots of different sizes are tasked with harvesting distinct lavender species, navigating passages of varying widths.

The problem assumes that the robots depart from their respective depots and return to the depots after visiting all the assigned tasks. Speed differences are reflected in the travel cost $cost_{ij}^{k}$, which represents the travel time for the robot *k* between locations of tasks *i* and *j*, using the average running speed of the robot. To incorporate kinematic feasibility, travel costs are computed by applying nonholonomic motion constraints. Travel costs are assumed to be symmetric between any two tasks. The robots are indexed k=1,⋯,m such that their speeds are non-increasing, meaning their travel costs are non-decreasing, i.e., $cost_{ij}^{1}\leq cost_{ij}^{2}\leq ,\cdots ,\leq cost_{ij}^{m},\;\forall i,j\in V_{k},\;k=1,\cdots ,m$.

The problem is formulated as a Mixed-Integer Linear Program (MILP), which is NP-hard [15]. The heuristics described in Section 3 are derived from the Linear Program (LP) relaxation of this model. Given *m* robots and *n* tasks, the sets, parameters, and variables used in the formulation are defined as follows:

Sets and indices:

$R=\{r_{1},\cdots ,r_{m}\}$

a set of robots

$D=\{d_{1},\cdots ,d_{m}\}$

a set of depots

$T=\{t_{1},\cdots ,t_{n}\}$

a set of tasks

$V_{k}=\left\{d_{k}\right\}\cup T$

a set of vertices for $r_{k}$

$E_{k}=\left\{\left(i,j\right),\;\forall i,j\in V_{k}\right\}$

a set of edges that connect all vertices in $V_{k}$

Parameters:

$P_{ij}$

the minimum width of passages where vertices *i* and *j* are located

$W_{k}$

the width of $r_{k}$
*M*
a sufficiently large constant (e.g., $\max(W_{k})$)

$cost_{ij}^{k}$

the travel cost of the edge from vertex *i* to *j* for $r_{k}$

$\delta_{k}\left(S\right)$

the subset of the edges of $E_{k}$ with one end in *S* and the other end in $V_{k}\setminus S$

Decision variables:

$x_{ij}^{k}$

the decision variable that represents whether edge (i,j) is used for the tour of $r_{k}$


$x_{ij}^{k}=\left\{\begin{array}{c} 1\;\mathrm{if}\;\mathrm{edge}\;\left(i,j\right)\;\mathrm{is}\;\mathrm{traveled}\;\mathrm{by}\;r_{k}\\ 0\;\mathrm{otherwise} \end{array}\right.$



$z_{U}^{k}$

the decision variable that represents the assignment of tasks in *T*


$z_{U}^{k}=\left\{\begin{array}{cc} 1 & \mathrm{if}\;U\;\mathrm{contains}\;\mathrm{all}\;\mathrm{vertices}\;\mathrm{not}\;\mathrm{assigned}\;\mathrm{to}\;r_{1},\cdots ,r_{k}\\ 0 & \mathrm{otherwise} \end{array}\right.$


*q*
a continuous variable representing the maximum travel cost (makespan)


The MILP formulation is presented below. Note that for the LP relaxation used by our heuristic, the binary constraints on $x_{ij}^{k}$ and $z_{U}^{k}$ are relaxed to be non-negative.

(1) $$\begin{array}{cc} C_{LP} & =\min q \end{array}$$

(2) $$\begin{array}{ccc} \;\sum_{\left(i,j\right)\in \delta_{1}\left(S\right)}x_{ij}^{1} & \geq 2-2\sum_{T⊇U⊇S}z_{U}^{1} & \;\forall S\subseteq T, \end{array}$$

(3) $$\begin{array}{ccc} \;\sum_{\left(i,j\right)\in \delta_{k}\left(S\right)}x_{ij}^{k} & \geq 2\sum_{T⊇U⊇S}\left(z_{U}^{k-1}-z_{U}^{k}\right)\; & \;\forall S\subseteq T,\;k=2,\cdots ,m-1, \end{array}$$

(4) $$\begin{array}{ccc} \sum_{\left(i,j\right)\in \delta_{m}\left(S\right)}x_{ij}^{m} & \geq 2\sum_{T⊇U⊇S}z_{U}^{m-1} & \;\forall S\subseteq T, \end{array}$$

(5) $$\begin{array}{cccc} \;P_{ij}-W_{k} & \geq M(x_{ij}^{k}-1) & \; & \;\forall \left(i,j\right)\in E_{k},k=1,\ldots ,m, \end{array}$$

(6) $$\begin{array}{ccc} \;q & \geq \sum_{i,j\in V_{k}}cost_{ij}^{k}\;x_{ij}^{k} & \;k=1,\cdots ,m, \end{array}$$

(7) $$\begin{array}{ccc} \;x_{ij}^{k} & \in \left\{0,1\right\},\;z_{U}^{k}\in \left\{0,1\right\},\;q\geq 0. & \; \end{array}$$

**Figure 2:**
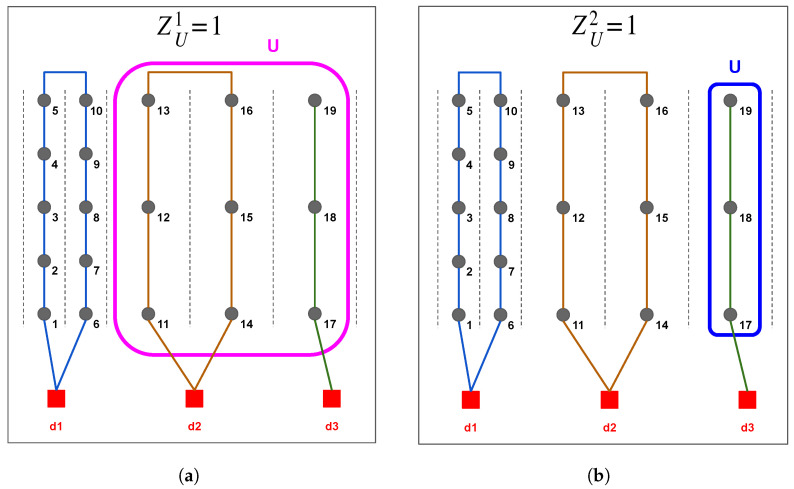
Example of the decision variable $z_{U}^{k}$ in a scenario with 3 robots and 19 tasks. (**a**) $z_{U}^{1}=1$ only if *U* includes all tasks not visited by the $r_{1}$, thus, when $U=\{t_{11},\cdots ,t_{19}\}$. (**b**) $z_{U}^{2}=1$ only if *U* includes all tasks not visited by $r_{1},r_{2}$, thus, when $U=\{t_{17},\cdots ,t_{19}\}$.

For any U⊆T, $z_{U}^{k}$ is defined for k=1,⋯,m−1. For the first robot, for any S⊆T, at least two edges must be selected from $\delta_{1}\left(S\right)$ for its tour if there exists at least one task in *S* that is not connected to any of the depots in the set $\left\{d_{2},\cdots ,d_{m}\right\}$; that is $\sum_{\left(i,j\right)\in \delta_{1}\left(S\right)}x_{ij}^{1}\geq 2$, if $\sum_{T⊇U⊇S}z_{U}^{1}=0$. This condition can be expressed as $\sum_{\left(i,j\right)\in \delta_{1}\left(S\right)}x_{ij}^{1}\geq 2-2\sum_{T⊇U⊇S}z_{U}^{1}$. Similarly, for any robot k∈{2,⋯,m−1}, for any S⊆T, at least two edges must be chosen from $\delta_{k}\left(S\right)$ for the tour of robot $r_{k}$ if *S* contains at least one task assigned to that robot. This constraint can be written as $\sum_{\left(i,j\right)\in \delta_{k}\left(S\right)}x_{ij}^{k}\geq 2\sum_{T⊇U⊇S}\left(z_{U}^{k-1}-z_{U}^{k}\right)$. Finally, for the last robot (k=m), the corresponding constraint is $\sum_{\left(i,j\right)\in \delta_{m}\left(S\right)}x_{ij}^{m}\geq 2\sum_{T⊇U⊇S}z_{U}^{m-1}$. Figure 2 provides an illustrative example of how the $z_{U}^{k}$ variable functions within the assignment structure.

Constraints (1) and (6) work together to minimize the makespan *q*, a standard technique to linearize a min–max objective. Constraints (2)–(4) are connectivity constraints that serve two critical functions: (1) they ensure the set of tasks assigned to all robots forms a complete partition of the total task set *T*, and (2) they act as subtour elimination constraints, ensuring each robot follows a single continuous tour. Constraint (5) is the explicit size constraint. Using the Big *M* method, it ensures that if a path (i,j) is used by $r_{k}$, then the condition $W_{k}\leq P_{ij}$ must hold. Consequently, nodes that are connected by passages narrower than $W_{k}$ are excluded from the feasible set of routes for robot $r_{k}$, preventing infeasible task assignments. Constraint (7) defines the domains for the decision variables.

## 3. Heuristic Approaches for Coordination of Heterogeneous Robotic Systems

This section details the three distinct heuristic approaches developed to solve the coordination problem for heterogeneous robotic systems: a primal–dual-based heuristic derived from the mathematical formulation, an intuitive greedy algorithm, and a novel approach utilizing a Large Language Model (LLM) through prompt engineering.

### 3.1. A Primal–Dual Based Heuristic

Our primal–dual heuristic is derived from the linear programming (LP) relaxation of the problem, as formulated in (1)–(4) and (6)–(7) while relaxing the size constraint (5). The algorithm leverages the duality of this LP relaxation to construct approximate solutions. The dual formulation is as follows:

(8) $$\begin{array}{ccc} \;C_{dual} & =\max2\sum_{S\subseteq T}Y_{1}\left(S\right) & \; \end{array}$$

(9) $$\begin{array}{ccc} \sum_{S:e\in \delta_{k}\left(S\right)}Y_{k}\left(S\right) & \leq cost_{ij}^{k}B_{k} & \;\forall i,j\in V_{k},\;k=1,\cdots ,m, \end{array}$$

(10) $$\begin{array}{ccc} \;\sum_{S\subseteq U}Y_{k}\left(S\right) & \leq \sum_{S\subseteq U}Y_{k+1}\left(S\right) & \;\forall U\subseteq T,\;k=1,\cdots ,m-1, \end{array}$$

(11) $$\begin{array}{ccc} \;\sum_{k=1,\cdots ,m}B_{k} & \leq 1 & \; \end{array}$$

(12) $$\begin{array}{ccc} \;Y_{k}\left(S\right) & \geq 0 & \;\forall S\subseteq T,\;k=1,\cdots ,m, \end{array}$$

(13) $$\begin{array}{ccc} \;B_{k} & \geq 0 & \;k=1,\cdots ,m. \end{array}$$

The core structure of the algorithm, including the iterative search and workload balancing mechanisms, is adapted from our previous work in [16]. The algorithm begins with all dual variables set to zero and increases them uniformly with fixed $B_{k}$ values. This process continues until a constraint in (9) or (10) becomes tight. If a constraint in (9) becomes tight, the corresponding edge is added to its respective robot’s graph $G_{k}$, merging the vertex sets it connects. If the newly formed set is reachable from the robot’s depot, it is marked as inactive; otherwise, it remains active. If a constraint in (10) becomes tight, the corresponding set is marked, and the iteration proceeds. The loop terminates when all sets for the first robot become inactive. The process is repeated for updated $B_{k}$ values to improve the workload balance. The primary modification in this work is the integration of Algorithm 1, which introduces a reallocation step to explicitly handle the size constraints after the initial relaxed assignment is determined. The following notations are used in the algorithm description:

Algorithm Notations:

$F_{k}$

Tasks allocated to $r_{k}$ while $F=\{F_{1},\cdots ,F_{m}\}$

$P_{k}$

Path of $r_{k}$ with tasks in $F_{k}$ while $P=\{P_{1},\cdots ,P_{m}\}$
*Z*
The set of violated assignments in the initial allocation

$G_{k}$

The graph that belongs to $r_{k}$

$N_{k}$

A queue of nearby depots for the $k^{th}$ depot arranged in ascending order


Algorithm 1 first identifies all task assignments that violate the size constraints and places them in a set *Z* for reassignment. It then iterates through each “violating” task z∈Z and evaluates the cost of reassigning it to every other robot capable of handling it. The reassignment that results in the minimum increase to the maximum cost (makespan) across all robots is selected. This ensures that all constraints are met while maintaining a balanced workload.
**Algorithm 1** [Tour, TourCost] = Reallocation (F)  1:**for** k=1,⋯,m **do**  2:    Let $Q_{k}$ be a set of vertices connected to $d_{k}$.  3:    **if** there exists any vertex that violates the size constraints **then**  4:        Add it to *Z* and remove it from $Q_{k}$.  5:    **end if**  6:**end for**  7:Remove all vertices in $Q_{1}$ from the rest of the graphs.  8:**for** ∀z∈Z **do**  9:    Find the robots that can handle *z*.10:     **if** there exists any target assigned to multiple robots **then**11:      **for** each $r_{k}$ that can handle *z* **do**12:          Form a temporary assignment by adding all vertices in the component connected          to *z* in $G_{k}$ to the $Q_{k}$ and removing them from the original assigned partition.13:          Compute the minimum spanning tree costs for the updated assignments and          save the maximum cost to compare.14:      **end for**15:      Compare the resulting costs and pick the lowest one.16:    **else**17:       Add all vertices in the component connected to *z* in $G_{k}$ to $Q_{k}$, and remove them       from other partitions if there are any.18:    **end if**19:**end for**20:Find the tour with the minimum cost for each $Q_{k}$.21:**return** Tour,TourCost

### 3.2. A Greedy Heuristic

In contrast to the formulation-driven primal–dual method, our second approach is a greedy heuristic based on intuitive, iterative improvement steps. The algorithm, detailed in Algorithm 2, consists of two main phases: initial routing and iterative redistribution. In the initial routing phase, the heuristic begins by generating a feasible initial solution. Each task is assigned to the robot that can service it with the lowest travel cost from its depot, without considering workload balance. Once all tasks are assigned, an optimal tour for each robot’s assigned task set ($F_{k}$) is calculated using an established solver. In our implementation, we use the Lin–Kernighan–Helsgaun (LKH) algorithm [17]. While this phase initially resembles classical nearest-neighbor heuristics, which assign tasks solely based on minimal travel cost, it goes beyond such methods by iteratively improving the solution to reduce the overall makespan (i.e., the maximum travel cost among all robots). The algorithm iteratively refines the initial solution until no further improvements are possible. In each iteration, (1) the robot with the current maximum travel cost, $r_{k}$, is identified as the most “overloaded”. (2) For each task in $F_{k}$, the algorithm identifies a set of candidate robots, $N_{k}$, that could potentially take over the task. These candidates are typically the robots with the second-lowest travel cost to that task. (3) The algorithm evaluates transferring each task from $F_{k}$ to a candidate robot in $N_{k}$. A transfer is executed only if it satisfies two conditions: (a) the transfer reduces the overall maximum travel cost, and (b) the new route for the recipient robot does not violate any size constraints. This process of identifying the most loaded robot and attempting to offload its tasks continues until no task transfer can be found that reduces the makespan; at this point, the solution is considered locally optimal. Our approach explicitly targets workload balance and route efficiency across heterogeneous robots, combining a simple initial assignment with a systematic, greedy improvement phase. This allows the algorithm to escape naive initial solutions and achieve higher-quality, balanced routes in practical scenarios.
**Algorithm 2** Coordination of multiple heterogeneous robots  1:Initial Routing  2:$F_{k}\leftarrow \emptyset$ for k=1,⋯,m  3:**for** j=1:n **do**  4:    Find a depot $d_{k}$ that could be reachable with the lowest cost and insert *j* into $F_{k}$.  5:**end for**  6:Find the optimal path $P_{k}$ with the tasks in $F_{k}$ for k=1,⋯,m.  7:Redistribution  8:**while** no exchange is available **do**  9:    κ={1,⋯,m}10:    **while** κ is not empty **do**11:        Find $r_{k}$ that has the maximum travel cost.12:        $\kappa \leftarrow \kappa \setminus r_{k}$13:        Find the neighboring depots $N_{k}$ by picking the depot with the second lowest cost        for all tasks in $F_{k}$.14:        **for each** depot $d\in N_{k}$ **do**15:            For all tasks in $F_{k}$, see if transferring the task to $r_{d}$ would decrease the maximum            travel cost while satisfying all the constraints, and transfer the task if possible.16:        **end for**17:     **end while**18:**end while**19:Find the optimal path $P_{k}$ with the updated $F_{k}$ for k=1,⋯,m.20:**return** $P_{k}$

### 3.3. A LLM-Based Heuristic via Prompt Engineering

While Large Language Models (LLMs) have shown versatility across many domains, their application to multi-robot coordination remains nascent. Recent studies have explored LLMs for robot action planning, but their methods often face challenges with scalability, real-time adaptability, and solution feasibility when applied to complex, heterogeneous routing problems. For instance, approaches like ProgPrompt [18] and LLM-Planner [19] can be limited by the need for real-time coordination or extensive training data. Similarly, frameworks such as SMART-LLM [20] may generate infeasible solutions, which is a critical issue for routing problems with strict constraints. Other works have used LLMs to find heuristics from existing solutions [21] or to improve success rates through self-debugging [22], but these methods do not always guarantee optimality or applicability to novel problems. These studies highlight a need for specialized approaches that can handle the unique constraints of dynamic, heterogeneous environments without relying on fine-tuning or iterative refinement.

To address these challenges, we propose a zero-shot, LLM-based heuristic that relies on carefully structured prompt engineering. Our methodology centers on designing a single, comprehensive prompt that provides the LLM with a complete definition of the environment, constraints, and optimization objectives. The goal is to enable the LLM to generate high-quality, feasible solutions without prior training on the specific problem type. This section details the prompt’s structured composition, which is key to achieving consistent and valid solutions.

The prompt is organized into three main sections:

- Role and goal definition: The prompt begins by explicitly instructing the LLM to act as an optimization expert. It emphasizes that all subsequent information must be carefully considered to generate a valid task allocation that respects all constraints and objectives.
- Zero-Shot Example (In-Context Learning): A complete, solved example is provided to illustrate the required logic and output format. This allows the LLM to learn from a single demonstration and generalize its reasoning to new instances. The example includes
  – Map information: Definitions of the coordinate system, traversable passages, and task locations.
  – Robot specifications: Depot locations and corresponding cost matrices for each robot, where infinite costs represent infeasible paths due to size constraints.
  – Constraints: An explicit explanation of how the cost matrices encode the size constraints.
  – Expected output: A sample solution showing the final task allocation for each robot.
- Problem Instance: Following the example, the prompt defines the new problem that the LLM must solve. This section mirrors the structure of the example but contains the specific map, robot, and task data for the target instance.

**Figure 3:**
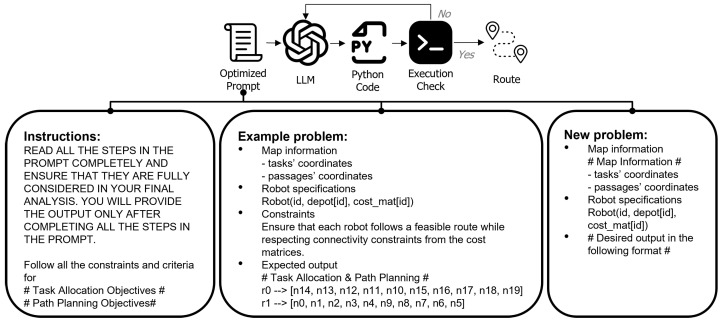
The flow of the LLM-based approach, showing how a structured prompt containing the problem definition and an example guides the LLM to produce a feasible solution.

This structured process, illustrated in Figure 3, is designed to provide the LLM with all necessary context—environmental, operational, and objective-based—to function as an effective zero-shot optimizer for this complex routing problem. Once the LLM generates a solution, a custom verification function checks that all tasks are assigned correctly and whether any constraints are violated. This mechanism ensures that infeasible solutions are corrected before final assignment. While our experiments show that this approach produces feasible solutions, infeasible outputs can still occur. In such cases, a refinement step can guide the LLM toward a valid solution. This underscores the necessity of precise prompt design, as well-structured and comprehensive prompts play a critical role in mitigating hallucinations in the LLM’s output.

## 4. Implementation and Computational Results

### 4.1. Experimental Setup and Performance Metrics

We validated the three proposed heuristic approaches through simulations on a PC with a 13th Gen Intel Core i7-1370P CPU and 64 GB of RAM. To ensure robust results, we generated 100 independent instances for each problem size by randomly placing tasks and depots within a given area. Each robot in the heterogeneous fleet was assigned a distinct average speed and width, with the travel cost, $Cost_{ij}^{k}$, defined as the travel time based on the robot’s average running velocity.

To evaluate performance, we assessed two key criteria: solution quality and computational time. Solution quality was measured with a Posteriori Bound (PB), which compares an algorithm’s makespan ($T_{algo}$) to a baseline’s makespan ($T_{baseline}$), calculated as $PB=T_{algo}/T_{baseline}$. A lower PB value signifies a better solution.

### 4.2. Analysis of Results

**Figure 4:**
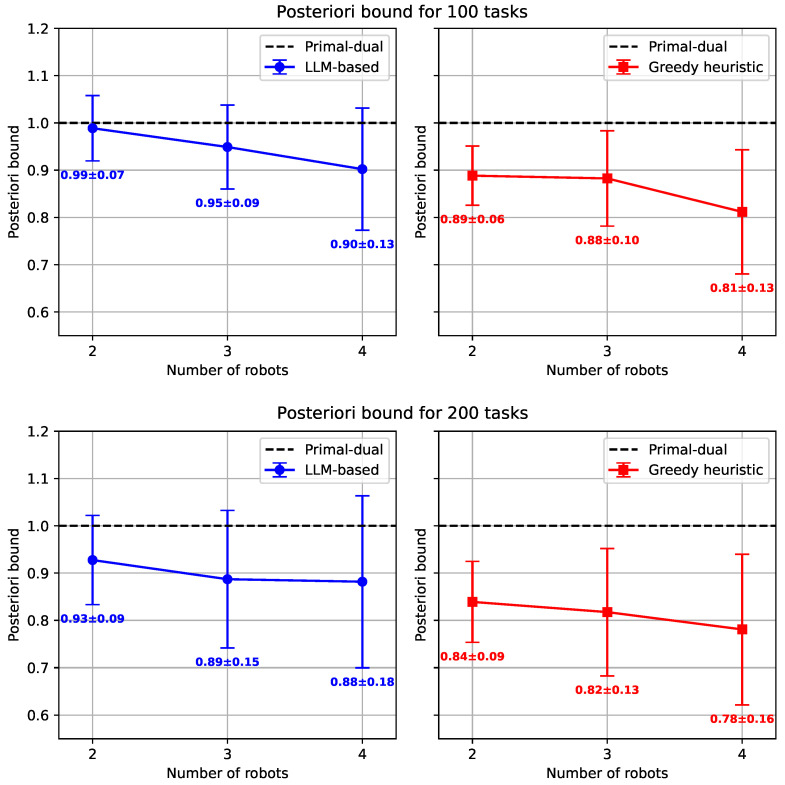
Posteriori bound for small-scale instances.

Our initial tests on small-scale problems (2–4 robots, 100–200 tasks) used the primal–dual heuristic as the baseline. As shown in Figure 4, both faster heuristics consistently outperformed the primal–dual approach on average. The greedy heuristic proved most effective, reducing the makespan by 11–19% for 100 tasks and 16–22% for 200 tasks compared to the baseline. The LLM-based heuristic also showed strong performance, with improvements of up to 10% for 100 tasks and 12% for 200 tasks. While the greedy heuristic achieved higher average performance than the LLM-based heuristic, the standard deviations indicate that both methods exhibited comparable variability across problem instances. This suggests that the LLM-based heuristic can be similarly reliable in terms of consistency, even though it occasionally produces less optimal solutions. The difference in computational efficiency, detailed in Table 1, is even more striking. The primal–dual heuristic’s runtime grew significantly with problem size, averaging over 100 s for 200-task instances. In contrast, the greedy and LLM-based heuristics were exceptionally fast, consistently returning solutions in sub-second times. Both heuristics demonstrated minimal runtime variability, indicating robust computational performance. Given its prohibitive computation time, the primal–dual approach was excluded from further testing on larger problem instances.

**Figure 5:**
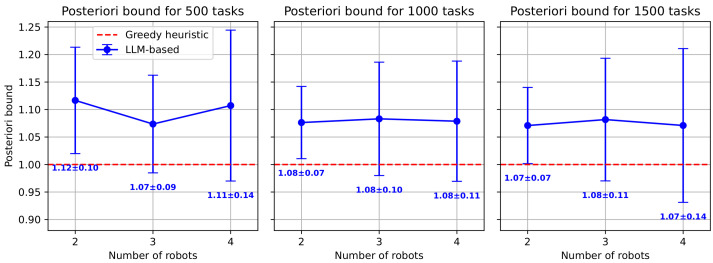
Posteriori Bound for larger-scale instances.

For larger-scale instances (up to four robots and 1500 tasks), we compared the greedy and LLM-based heuristics, using the superior greedy algorithm as the new baseline. Figure 5 shows that the LLM-based heuristic produced solutions of lower quality, with an average makespan that was 7–12% worse than the greedy approach. An analysis of the computational time, detailed in Table 2, further reinforces the superior performance of the greedy heuristic. The greedy algorithm remained computationally efficient, with its maximum runtime peaking at 5.3 s even for the largest 1500-task instances, and the low standard deviations reflect highly predictable performance. In contrast, the LLM-based heuristic’s runtime scaled significantly with problem complexity, requiring up to 16.3 s on average and reaching a maximum of 21.8 s for the 1500-task, four-robot scenario. However, its comparable runtime variance demonstrates that, despite longer runtimes, the computational effort of the LLM-based heuristic is consistent. These results demonstrate that while the LLM can generate feasible solutions for large-scale problems, the greedy heuristic offers both faster computation and higher-quality solutions, making it the more effective approach for these complex instances.

## 5. Conclusions

This paper addressed the complex challenge of coordinating heterogeneous robotic systems in agriculture by developing and comparing three distinct heuristic approaches: a primal–dual algorithm, a specialized greedy heuristic, and a novel method using an LLM with zero-shot prompt engineering.

Our computational results demonstrated that for the specific problem of minimizing makespan under size constraints, the greedy heuristic provided the most optimal and computationally efficient solutions at the current scale, as it is specifically designed for the current problem formulation. Its performance advantage arises from a simple initial assignment combined with iterative redistribution, though more complex problems may require improved initial routing to achieve efficiency. In contrast, the LLM-based heuristic represents a significant advancement in flexibility and adaptability. Without task-specific fine-tuning, it produces high-quality, feasible solutions and can be relatively easily modified to accommodate new, complex tasks that incorporate robot-specific capabilities or adapt to dynamic environmental changes by adjusting the natural language prompt. While its average performance was slightly lower and its runtime was higher than the greedy heuristic, its ability to handle complex and evolving problem definitions makes it particularly valuable in scenarios requiring adaptability. This achievement showcases the potential of LLMs to function as generalized problem-solvers in robotics. This is critical for real-world agricultural environments where operational requirements can change daily. The ability to generate valid solutions from a high-level description dramatically lowers the barrier for developing and deploying robotic coordination strategies.

Future work will focus on two primary directions. First, we aim to enhance the LLM-based approach by exploring more advanced techniques, such as chain-of-thought prompting or allowing the model to interact with external code libraries and optimization tools, which could improve its performance and reliability. Second, we will focus on bridging the gap in the real-world deployment of heterogeneous robots by extending the problem formulation to incorporate practical operational constraints and environmental uncertainties—such as heterogeneous robot capabilities, battery and payload limits, dynamic obstacles, terrain variations, and task dependencies. Validating these advanced algorithms through physical experiments on a robotic fleet will be a crucial next step toward deploying autonomous systems in dynamic agricultural environments.

## Figures and Tables

**Table 1:** Computation time and standard deviation for small-scale instances.

# of Robots	100 Tasks			200 Tasks		
	Primal–Dual	Greedy	LLM-Based	Primal–Dual	Greedy	LLM-Based
Average computation time in seconds						
2	11.25	0.01	0.04	86.96	0.02	0.14
3	13.20	0.02	0.05	97.09	0.04	0.20
4	15.16	0.03	0.07	103.06	0.07	0.29
Maximum computation time in seconds						
2	38.80	0.01	0.20	224.73	0.05	0.24
3	40.18	0.04	0.20	235.32	0.09	0.30
4	46.23	0.05	0.23	217.34	0.13	0.55
Standard deviation of computation time						
2	3.71	0.00	0.02	21.62	0.01	0.02
3	6.27	0.01	0.02	39.04	0.02	0.03
4	7.01	0.01	0.02	27.47	0.02	0.07

**Table 2:** Computation time and standard deviation for larger-scale instances.

# of Robots	500 Tasks		1000 Tasks		1500 Tasks	
	Greedy	LLM-Based	Greedy	LLM-Based	Greedy	LLM-Based
Average computation time in seconds						
2	0.08	0.90	0.26	3.60	0.56	8.27
3	0.18	1.38	0.55	5.80	1.24	12.23
4	0.28	1.79	0.88	7.26	1.74	16.26
Maximum computation time in seconds						
2	0.19	1.14	0.94	4.16	1.60	9.11
3	0.55	2.02	2.08	8.73	3.12	14.55
4	0.58	3.43	2.35	10.12	5.35	21.79
Standard deviation of computation time						
2	0.03	0.90	0.17	0.21	0.34	0.27
3	0.09	0.25	0.32	0.95	0.69	1.41
4	0.11	0.33	0.44	1.16	0.92	2.60

## Data Availability

The original contributions presented in this study are included in the article. Further inquiries can be directed to the corresponding authors.
